# Microfinance for women at high risk for HIV in Kazakhstan: study protocol for a cluster-randomized controlled trial

**DOI:** 10.1186/s13063-018-2566-y

**Published:** 2018-03-20

**Authors:** Tara McCrimmon, Susan Witte, Gaukhar Mergenova, Assel Terlikbayeva, Sholpan Primbetova, Azamat Kuskulov, Scarlett L. Bellamy, Nabila El-Bassel

**Affiliations:** 1 Global Health Research Center of Central Asia, Almaty, Kazakhstan; 2 Columbia University of Social Work, 1255 Amsterdam Ave, New York, NY 10027 USA; 3 Dornsife School of Public Health, Drexel University, Philadelphia, PA USA

**Keywords:** Women, HIV, STI, Drug use, Microfinance

## Abstract

**Background:**

Among women at high risk for HIV and other sexually transmitted diseases (STIs), gender and economic issues limit the impact of behavioral prevention strategies. Women in Kazakhstan with dual risks of sex trading and drug use face elevated risk for HIV and STIs and may benefit from an economic empowerment intervention which combines HIV-risk reduction (HIVRR) education with financial skills-building and asset-building to promote reduced reliance on sex trading for income.

**Methods/design:**

The study employs a two-arm, cluster-randomized controlled trial (c-RCT) design. We will use cluster randomization to assign 350 women in approximately 50 cohorts to a traditional four-session HIV-risk-reduction intervention combined with a six-session financial literacy intervention, enrollment in a 24-session vocational training program and receipt of matched savings (HIVRR+MF); or to the four-session HIV-risk-reduction intervention alone (HIVRR). Repeated behavioral and biological assessments will be conducted at baseline, then at 6, 9, and 15 months post randomization/session 1.

**Discussion:**

This study responds to an identified need in the academic literature for rigorous testing of structural interventions, including combination microfinance and HIV-prevention interventions.

**Trial registration:**

ClinicalTrials.gov, ID: NCT02406482. Registered on 30 March 2015.

**Electronic supplementary material:**

The online version of this article (10.1186/s13063-018-2566-y) contains supplementary material, which is available to authorized users.

## Background

While the HIV epidemic in Kazakhstan has remained concentrated among high-risk populations since its beginnings, a recent shift from parenteral to heterosexual transmission highlights the increased risk of HIV transmission to, and by, female sex workers (FSW). HIV prevalence continues to rise among FSW throughout the Central Asian region, yet there has been limited research and exposure to evidence-based HIV-prevention interventions for this population [1]. As of 2014, there were an estimated 19,600 FSW in Kazakhstan, yet only 15 of 93 registered HIV service non-governmental organizations (NGOs) throughout the country served FSW [2]. One systematic review and meta-analysis has estimated that in low- and middle-income countries like Kazakhstan, sex workers have 13.5 times the odds of contracting HIV compared to all women of reproductive age [3]. While official estimates put the prevalence of HIV among FSW in Kazakhstan at 1.3% [4], HIV prevalence is higher among FSW who use both injection and non-injection drugs than among FSW who do not share these additional risks [1]. Drug-using female sex workers are at the greatest HIV risk and the least likely to have access to testing, care or treatment services – they operate at the lower end of the market, are more often street-based, and are typically much poorer than non-drug-using FSW [2]. These women’s low social and economic standing may not only drive entry into sex work, but may make them financially dependent on paying or intimate male partners for living and drug expenses, less able to negotiate safe sex or safe injection practices, and may increase their risk for physical or sexual violence [5–7]. Stigma against both sex work and drug use and aggressive policing may limit their access to health services including drug treatment as well as legal recourse, and may subject them to sexual coercion and violence from police themselves [6, 8]. Furthermore, in Central Asia, few women who use drugs have been reached through evidence-based HIV or harm-reduction interventions, and most local HIV or drug treatment programming has not met their unique needs [9].

Given how these risks are rooted in women’s low economic and financial standing, promoting women’s economic empowerment may provide structural protection from HIV. Structural approaches to HIV prevention are characterized by Gupta et al. (2008) as those which address the physical, social, economic, and policy environments which create vulnerability to HIV [10]. This includes programs which seek to alleviate the economic inequalities faced by high-risk women [11–13]. “Microfinance” has been identified as an important structural intervention for HIV prevention and improved access to testing, care, and treatment [13–15]. The term microfinance covers an array of programs and policies, including financial literacy education, vocational training, conditional or unconditional cash transfers, formal or informal microcredit or lending, small business development, and asset-building through savings programs. In Kazakhstan, microfinance played a crucial role in the establishment of private enterprise after the fall of the Soviet economic system [16]. Microfinance programs usually include a collaboration between a banking institution and an NGO or United Nations agency, offering business guidance such as product development and marketing in combination with a microloan [16]. However, we found no microfinance programs that either target or incorporate drug users or sex workers. Microfinance lenders may consider these risk groups too unstable or unreliable for loans, and stigma against these risk groups may lead to them being overlooked for other forms of microfinance programming.

There is a small but growing body of empirical studies done worldwide which have sought to demonstrate the impact of microfinance interventions on HIV prevention and care [14, 15, 17–20]. These studies have utilized a range of the microfinance strategies listed above, either introducing them individually or in combination with behavioral interventions for HIV-risk reduction (HIVRR). Several studies targeted FSW, including Project JEWEL in Baltimore, which provided training in craft-making to FSW who used drugs, and showed, on average, a decrease in the number of both sexual contacts and paying partners, as well as increased condom use [18]. Through a similar study in Chennai, India, FSW were taught tailoring skills, and saw a decrease in paying partners, as well as increased licit income [19]. A microenterprise intervention with FSW in Kenya’s urban slums, which offered financial literacy training and microloans, demonstrated an overall decrease in the number of sex partners and a higher consistency of condom use; furthermore, nearly half of participants stopped sex work altogether and two thirds developed operational businesses [20].

The majority of microfinance studies included a microcredit or loan component (both group and individual loans) to support small business development, or a vocational training component, but few have focused on asset-building. The Undarga study among street-based sex workers in Ulaanbaatar, Mongolia, is the first to have addressed this, using a randomized controlled trial (RCT) design to test the efficacy of a savings-led microfinance program in combination with HIVRR [17]. The benefit of savings-led interventions over microcredit interventions is that they enable participants to accumulate assets faster and pay for life events without accumulating debt and an over-reliance on microloans, for which interest rates can be onerous. This makes them less likely to cause additional financial strains for populations like FSW, which already face significant financial burdens. Participants in Undarga were randomized to receive either a four-session HIVRR intervention alone, or the same four-session HIVRR intervention and a savings-led microfinance program. The microfinance components included financial literacy training, a business development training with mentorship, and a matched savings program to help women establish small businesses. Findings showed that women in the microfinance arm reported significantly fewer unprotected sex acts at 6 months post intervention and that women in the microfinance arm reported a significantly lower percentage of income from sex work, increased odds of reporting no income from sex work, and increased odds that sex work was not their main source of income at 6 months post intervention compared to women who received HIV prevention alone [17, 21]. While women from both study arms reported a decrease in the number of paying sexual partners between the baseline and 6 months post-intervention assessments, those who had received the microfinance components reported a significantly larger decrease than those who received HIVRR alone [17].

While this study suggests the success of savings-led microfinance for HIV-risk reduction among FSW, gaps remain in the evidence base for microfinance for HIV-risk reduction among women who use drugs. The four studies detailed above are the only ones which have targeted female sex workers, and only one of these three targeted FSW who used drugs. Furthermore, past studies have assessed a limited range of outcomes, mostly self-reported sexual risk behaviors. No studies to date have used biological measures of HIV or other STIs in their assessments, and only one considered ART adherence as an outcome. Finally, only a few of these studies utilized a RCT design.

This paper presents the protocol for a study which tests a combination HIVRR and savings-led microfinance intervention. The study described here is adapted from the Undarga study protocol [17]. It responds to calls for more structural interventions for HIV focused on women who use drugs [22] and rigorous testing of HIVRR economic empowerment interventions through a RCT [13]. To the best of our knowledge, it is the only study to date to incorporate biological assessments, including testing for HIV and other STIs, and viral-load and CD4 biomarkers for those who are HIV positive. It is one of the few interventions that focuses on the key affected population of women at dual risk of HIV from both drug use and sex work. This study should have global as well as regional implications for structural interventions for HIVRR.

## Methods/design

This study uses a cluster randomized controlled trial (c-RCT) to evaluate a combination HIVRR and microfinance (MF) intervention among FSW who use drugs in Kazakhstan. The study compares two arms: a combination HIVRR+MF intervention treatment arm and a control arm, which receives the HIVRR component alone. The study assesses outcomes over a 15-month period, which encompasses up to 3 months of intervention activities, and 12 months of follow-up. Study recruitment and enrollment began in May 2015, and follow-up data collection will be completed in October 2018.

**Fig. 1:**
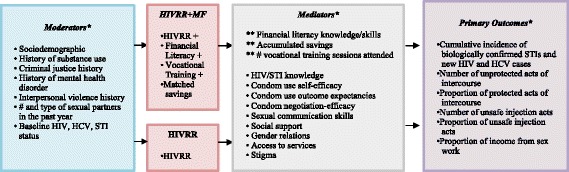
Theoretical framework

The theoretical framework for the study is shown in Fig. 1. The primary aim of the study is to test whether participants assigned to the combined HIVRR+MF condition have improved outcomes on a number of indicators, as compared to those assigned to the HIVRR condition. These indicators include: (1) a lower cumulative incidence of biologically confirmed STIs (syphilis, gonorrhea, trichomoniasis, chlamydia and mycoplasma), (2) a lower rate of new HIV and hepatitis C virus (HCV) cases, (3) a greater decrease in the number of unprotected vaginal and anal sexual acts and a greater increase in the proportion of protected sexual acts with both regular and paying partners, (4) a reduction in the proportion of unsafe injection acts for those participants who inject drugs, and (5) a lower proportion of monthly income from sex work. These outcomes are summarized in Table 1.

**Table 1:** Assessment variables

	Measurement	Timepoint^a^			
Moderators		B	M6	M9	M15
Age, income, education, marital status, experience of sex work, trauma	Sociodemographic Questionnaire	X	X	X	X
Substance abuse and criminal justice history	Modified Risk Behavior Assessment (RBA) [36]	X	X	X	X
Mental health status	BSI [37]	X	X	X	X
Partner violence history (paying and intimate)	Revised Conflict Tactics Scale [38]	X	X	X	X
Number and type of sexual partners (past year)	Modified RBA [36]	X	X	X	X
Perceived stigma	Modified Sex Worker Stigma (SWS) Index [39]	X	X	X	X
Mediators		B	M6	M9	M15
HIV/STI knowledge	HIV/STI knowledge [40]	X	X	X	X
Condom use self-efficacy	Condom Use Self-Efficacy Scale [41]	X	X	X	X
Condom use outcome expectancies	Condom Barriers Scale [42]	X	X	X	X
Condom negotiation self-efficacy	Condom Negotiation Self Efficacy Scale [43]	X	X	X	X
Sexual communication skills	Sexual Communication Scale [44]	X	X	X	X
Social support	MSPSS [45]	X	X	X	X
Current partner violence (paying and intimate)	Revised Conflict Tactics scale [38]	X	X	X	X
Access to services	RBA Services [36]	X	X	X	X
^b^Financial literacy	Financial literacy knowledge [46]	X	X	X	X
^b^Savings deposits	Bank Statements		X	X	X
^b^Vocational training sessions attended	Attendance/process		X	X	X
Outcomes		B	M6	M9	M15
STI (gonorrhea, syphilis, trichomoniasis, chlamydia, *Mycoplasma genitalium*), HCV, and HIV status	Laboratory assays (see Table 2)	X		X	X
Sexual behavioral risk: number and % of unprotected sexual acts	Modified RBA [36]	X	X	X	X
Number and % shared injection acts, other drug use	Modified RBA [36]	X	X	X	X
Proportion of income from sex work, savings, debt	Economic Indicators Questionnaire	X	X	X	X
Cost of staff time, supplies, overhead for HIVRR and for MF	Project records; Administration review	Ongoing			

^a^*B* baseline (prior to randomization or intervention); *M6* month-6 follow-up, *M9* month-9 follow-up, *M15* month-15 follow-up

^b^Uniquely associated with asset theory and the HIVRR+MF arm

*HCV* hepatitis C virus, *HIV* human immunodeficiency virus, *HIVRR* HIV relative risk, *MF* microfinance, *STI* sexually transmitted disease

Secondary aims of the study include an examination of how study outcomes are moderated by personal and structural characteristics, including sociodemographics, substance use history, criminal justice history, sexual history, etc. Secondary aims also include an examination of how study outcomes are mediated by several key, theory-driven variables, such as financial literacy knowledge and skills, HIV knowledge, condom use self-efficacy, se outcome expectancies, etc. In addition, a qualitative component will examine women’s personal reactions to each of the HIVRR+MF components, factors promoting the use of the intervention, and barriers that may impede their participation and the fidelity of intervention implementation. Finally, a cost-effectiveness component is included to estimate the costs and cost-effectiveness of the HIVRR+MF intervention on averted cumulative STIs, HCV, and HIV over the 12-month period.

### Study setting

This study is being conducted in two cities in Kazakhstan: Temirtau, and Almaty. These two cities are geographically distant from one another, with distinct characteristics. Almaty is the largest city in Kazakhstan (population 1,703,500) and its economic center [23]. Located close to the country’s southern border with Kyrgyzstan, it attracts both internal and external migrant workers through trade and job opportunities. Temirtau (population 181,197) is an industrial city in northern Kazakhstan [24]. The city is known for high rates of injection drug use in the years following the collapse of the Soviet Union. This study utilizes a staggered site start plan; recruitment began in Temirtau in May 2015 and in Almaty in February 2016. A project field office was established in each city where both assessment and intervention activities are conducted.

### Participant characteristics

Study participants are women who report recent histories of both drug use and sex trading. We plan to enroll a total of 350 women between the two study sites. In order to be eligible for the study, participants must: (1) be over 18 years old; (2) report illicit drug use within the past 12 months; (3) report having provided sex in return for money, goods, drugs or services within the past 90 days; and (4) report at least one incidence of unprotected sex (with either a paying or non-paying partner) within the past 90 days. Participants are ineligible if they (1) cannot communicate in Russian; (2) intend to move from the study site within the following 12 months, or (3) are deemed to have cognitive impairment that would affect their ability to provide consent or participate fully in the intervention activities.

### Intervention

All participants receive four HIVRR sessions and those assigned to the combination HIVRR+MF treatment arm receive an additional 30 sessions, including (1) six training sessions focused on financial literacy, (2) 24 sessions of vocational training in hairdressing, sewing, or manicurist professions, as well as (3) a matched savings program which incentivizes them to accumulate assets for small business development or job/vocational training. The NOVA intervention period lasts a total of 3 months post randomization, with intervention activities lasting for 2 weeks in the control arm, and for the full 3 months in the treatment arm. A brief description of each intervention component is provided below:

#### HI- risk reduction (HIVRR)

During the first 2 weeks of the intervention period, both study arms receive the HIVRR intervention. The HIVRR intervention was adapted from the Women on the Road to Health (WORTH) intervention [25]. The CDC identified WORTH as a best-practice, evidence-based intervention [26] and it has been implemented in a number of studies in the USA with women who use drugs [25, 27]. We have adapted the core components of WORTH to meet the cultural context and experience of women who use drugs and engage in sex work in Kazakhstan. The HIVRR intervention is guided by social cognitive theory [28]. It focuses on both sexual and drug use risk reduction, and is designed to increase communication, problem-solving skills, and self-efficacy related to safe-sex behaviors and drug use. Participants explore how drug use impacts their sexual risk with both paying and intimate partners, and are trained in communication skills with both. Role play activities provide the opportunity for women to practice these skills and to identify and problem-solve barriers to applying them. Participants undergo several intimate-partner violence (IPV) safety planning exercises, and regular check-ins by session facilitators ensure that women receive support and referrals if they feel that their safety is being threatened by an intimate partner or by another person during the course of the study. Participants receive basic training in emergency overdose response skills. Participants discuss informal and formal support systems available in their community. Linkage to HIV care, drug treatment (where available) and other services are provided throughout their enrollment in the study. Each session has goals setting and homework assignment that provide the opportunity for the women to practice the skills that they have learned. Participants receive HIVRR in a four-session intervention delivered twice a week for 2 weeks. Sessions are conducted in Russian by trained female facilitators and last for approximately 2 h. Participants receive small financial incentives (US$12/session) for sessions attended, as well as small safe-sex kits of condoms and lubricant.

#### Financial Literacy Training

During weeks 3 and 4 of the intervention period, the treatment arm only receives Financial Literacy Training (FLT). The FLT intervention was adapted from the financial literacy intervention used in the Undarga study [17]. FLT sessions focus on facilitating access to banking services (including how to open an account and use an ATM card), household budgeting (including development of a household budget), short- and long-term savings, debt management and financial negotiations. Sessions are designed to increase participant knowledge, skills, and self-efficacy related to managing and controlling finances, which might enhance their capacity for savings. Participants continue to build upon the goal-setting skills that they developed in the HIVRR sessions, and facilitators continue to conduct safety check-ins. Sessions are held three times per week over 2 weeks for a total of six sessions. Sessions are conducted in Russian by trained female facilitators, and last for approximately 2 h. Participants receive small financial incentives (US$12) and safe-sex kits for sessions attended.

#### Vocational Training (VT)

During months 2 and 3 of the intervention period, the treatment arm receives the Vocational Training (VT) intervention component. VT provides technical education and skills-building in a craft or trade. Including VT as a component in the HIVRR+MF intervention arm allows participants to gain competence in an employable trade, which grounds the intervention in the real world for scale-up after the trial ends. Participants in the treatment arm decide whether they wish to be enrolled in hairdressing, sewing, or manicurist vocational training sessions (dependent on course availability at each study site). Our selection of these options was informed by local feedback and a feasibility pilot conducted prior to study start, which confirmed that these were considered growing small business targets for women and that high-risk women reacted favorably to this model of vocational training. These trainings are conducted by professionals, who work either individually in the community or through a school or training program. VT partner sites were selected based on the quality of their facilities, the availability of afternoon and part-time training hours to accommodate participant schedules, and a reputation for successful graduation and certifications among students. All treatment arm participants are eligible to attend VT sessions regardless of how many FLT sessions they attended, as the material presented in the VT sessions is unrelated to the FLT. Vocational training is provided three times per week over the course of 2 months, for 24 sessions. Tuition is free to participants and women attend in small groups with only their cohort peers. Participants receive small financial incentives (US$12) for sessions attended.

#### Matched savings

From week 3 until the end of the intervention period at the end of month 3, the treatment arm receives the matched savings component of the intervention. The matched savings component of the HIVRR+MF intervention is also adapted from the Undarga program [17]. Building assets prior to shifting sources of income or moving to new or self-employment may increase the likelihood for increased income from other sources than sex work. As mentioned above, participants receive incentive payments (US$12/session) for each FLT or VT session attended. During the FLT intervention, participants receive support to open a personal savings account in a partner bank, and are encouraged to deposit their session attendance incentives in this account. Throughout the rest of the treatment intervention period, for every dollar of their incentive payment money deposited in their personal account, the project will deposit an equal amount in a separate, project-controlled account under the participant’s name. Participant’s funds will only be matched up to the total amount that they have received from incentive payments for session attendance – this limit ensures that women are not earning income through sex work for their matched savings. If a participant decides to save 100% of her incentive after every session, she will have saved a total of US$360 and would receive a matching US$360 for a total of US$720. Due to lack of documentation, lingering distrust of the banking system, or other barriers, some women may elect to never open a bank account. These participants have the option to store money with the program staff in an informal account until the time of withdrawal, and these informal accounts are also matched in a separate, project-controlled account under the participant’s name.

For a duration of 6 months after the duration of intervention sessions (until the month-9 follow-up assessment) participants may use matched savings amounts to pay for business or education expenses, including equipment and supplies for hairdressing, sewing, or manicurist professions (e.g., rental of a chair in a salon, products, fabric, sewing machines, etc.) or additional training (e.g., extra course fees). FLT intervention facilitators conduct a final “transitional session” at the end of the vocational training courses where participants are asked to set long-term goals regarding their chosen profession, and plan for spending their accrued matched savings. FLT intervention facilitators work with each participant to identify ways in which she can spend her matched savings. When a participant is ready to make a vocation-related purchase, she must provide half the amount of this purchase from her personal savings. The other half the amount of the purchase is provided by project staff from the participant’s matched account directly to the vendor or educational establishment. In order to receive this 1:1 match on their purchases, participants are required to make these purchases within 6 months of completing their vocational training (until their month-9 follow-up assessment); after this time, the project will not provide half the payment.

#### Retention sessions for control participants

As the treatment arm spends approximately 10 weeks more in the intervention sessions than the control arm, we utilize retention visits among the control cohorts to minimize a potential attentional-effect bias in favor of the treatment groups. These retention visits occur once a month for 3 months following the HIVRR intervention. Control arm participants are invited back to the field office together as a cohort, to receive small gifts (phone cards and toiletries) and to provide updated contact information to project staff. No additional training or intervention activities are provided at these retention visits, but participants are given the opportunity to interact with one another and discuss the content of the HIVRR sessions.

#### Intervention facilitators and supervision

The HIVRR and FLT sessions are led by trained study facilitators, supervised by study staff and investigators. A number of quality assurance measures have been put into place to ensure standardization across sessions and facilitators. Facilitators complete session evaluation forms after each session. Sessions are audio-recorded, and tapes are randomly selected for review by local clinical supervisors and US-based study investigators. Clinical supervisors note adherence to the intervention protocol and address any areas of immediate concern directly with the facilitators. Team supervision is held once a month with US-based study investigators through Skype calls to discuss the challenges faced by facilitators in conducting the sessions and adhering to the protocol.

### Study procedures

**Fig. 2:**
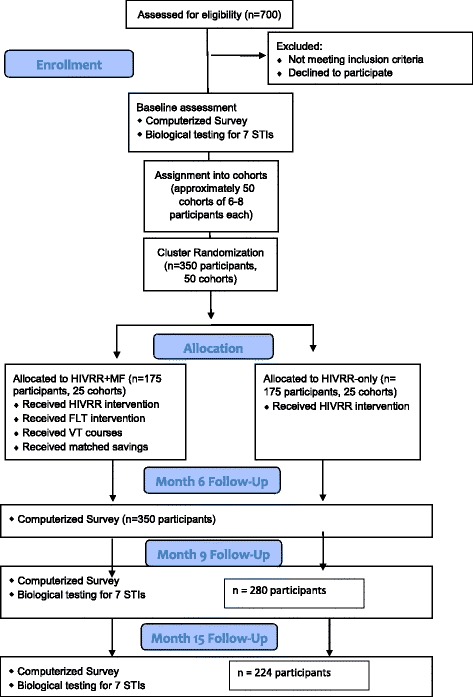
Consolidated Standards of Reporting Trials (CONSORT) flow diagram

**Fig. 3:**
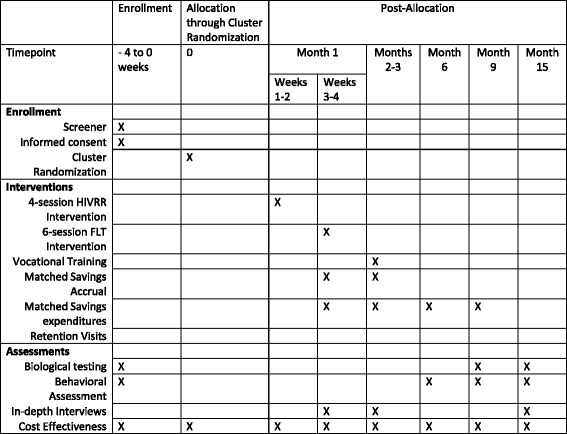
Standard Protocol Items: Recommendations for Interventional Trials (SPIRIT) schedule

The study design is summarized in the Consolidated Standards of Reporting Trials (CONSORT) diagram (Fig. 2) and in the Standard Protocol Items: Recommendations for Interventional Trials (SPIRIT) schedule (Fig. 3 and Additional file 1). We expect to enroll 350 participants in the intervention over the course of 2 years, and to follow them up over 15 months post randomization. Prior studies in Kazakhstan have yielded retention rates of 88% [29]; we expect to retain 80% (*n* = 280) of participants at their month-15 follow-up assessment.

#### Start-up phase activities

During the start-up phase of the study, study research assistants identified a number of field locations from which to recruit potential participants. These included hotels, saunas, and other locations where sexual services are known to be offered, medical organizations such as local AIDS centers and drug treatment clinics, and NGOs providing HIV support or social services to women. Research assistants observed these sites to uncover patterns in availability of potential participants, dependent on season, day of the week, holidays, etc. During this exploratory phase, they also noted community-based “gatekeepers” who control access to potential sex workers. These not only included the NGOs and outreach workers who were our collaborating partners, but also those who manage sex work transactions in the community. While we discovered that there are very few formal “pimps” or “madams” who run official sex work establishments, sex workers in Kazakhstan often work through local hotel and sauna administrators, who direct clients to them. Research assistants described the study to these gatekeepers as a project meant to improve the health of sex workers, emphasizing the benefits of HIV and STI testing that it would provide, and asked for their help in referring potential participants when recruitment began. The relationships established through this phase reduced resistance and uncertainty from these community gatekeepers, easing later recruitment efforts.

#### Recruitment and screening

This study utilizes two channels of participant recruitment: field recruitment visits by research assistants, and network-based recruitment through a peer-referral program. Research assistants conduct regular visits to NGOs, HIV clinics, drug treatment clinics, hotels, saunas and street-based sex work venues, where they approach potential participants, provide them with a brief overview of the intervention, and then ask for their verbal consent to conduct a screening for study eligibility. To extend recruitment deeper into communities of FSW, eligible participants receive a small financial incentive (US$5) to refer their friends and acquaintances to be screened for the study. This is done through a system of paper coupons. The referred network members contact the project staff and come to the project field office location at their convenience to complete screening.

A computerized survey tool is used for eligibility screening procedures. Research assistants read the computerized questionnaire aloud to participants, asking them a number of demographic and risk-behavior questions, including those described in the eligibility section above. Participants are compensated US$1 for their time. The survey tool automatically calculates whether participants are eligible based on the responses entered by the research assistant. If participants are not eligible for any reason, they are invited to be screened again after 90 days have passed.

Eligible participants are scheduled for study intake procedures and the baseline assessment (described below) at the project field office location as soon as possible after the screening. During this baseline visit, research assistants describe the details of the study to potential participants and lead them through an informed consent process.

Research staff enroll each participant into an intervention cohort within 2 weeks of their baseline. If more than a month has passed between the participant’s baseline survey and the start of the intervention session, the participant is re-screened to confirm continued eligibility, and baseline assessments are conducted again to collect more recent data.

#### Group assignment and randomization

We enrolled participants into groups of six to eight participants each, then randomized and followed them as a cohort over time. Together as a cohort, participants are randomized to either the treatment (HIVRR+MF) or control (HIVRR) condition at the time of the first intervention session. Randomizing by cohort is more efficient than attempting to accrue enough women in a short enough period to randomize individually and simultaneously to two conditions. A random-number generator randomly assigned each cohort of participants to either the treatment or control condition. This random number generator ensured a balanced assignment of cohorts to each arm over time. Neither participants nor study staff are blind to study condition.

#### Process measures

In addition to the intervention quality control measures described above, a number of process evaluation measures are collected by the study staff to ensure fidelity and standardized delivery of study components among participants and between two diverse study sites. Among the process evaluation measures included are intervention session attendance (for both treatment and control arms), vocational training attendance (for treatment arm) and matched savings deposits and spending (for treatment arm), and retention measures. These process measures are used for a number of reasons, including supervision, assessment of standardization and fidelity to the intervention, and for use in booster training events throughout the study period.

#### Assessments

Baseline and follow-up assessments are administered by research assistants in project field office locations. Assessments take place at a pre-intervention baseline (prior to cohort assignment and randomization), then at 6, 9, and 15 months after randomization and intervention session 1 (which occur simultaneously, as participants are randomized as a cohort immediately prior to beginning the first intervention session). Since the HIVRR+MF treatment arm receives an intervention that is approximately 2.5 months longer than the control arm, we allocated an additional 2.5 months of follow-up time for in the control arm. Behavioral questionnaires are utilized at all assessments, and biological testing procedures are conducted at baseline, month-9 and month-15 assessments.

Behavioral questionnaires are conducted using computer-assisted self-interviewing (CASI) methods, with the same survey tool that the research assistants use to assess study eligibility and which can be completed in approximately 2 h. The baseline behavioral questionnaire addresses the main outcome variables of interest to the study (number of partners, number of sex acts, number of sex acts which are unprotected, number of paying partners, incidences of drug use, incidences of injection drug use and incidences of risky injection behaviors) in addition to several of the theoretical constructs underlying the study (self-efficacy in a number of risk-reduction behaviors, outcome expectancies and risk-reduction intentions) and a number of potential moderators (demographics, financial status, experience of IPV and gender-based violence (GBV), social support, linkage to care and service utilization). Table 1 contains a list of the specific measures used for each construct.

Follow-up assessments occur as early as 1 week before the exact follow-up date, and as late as 3 weeks after the exact follow-up date. Like the baseline behavioral questionnaire, the follow-up behavioral questionnaires address the main outcome variables, moderators and mediators of interest to the study. The follow-up questionnaires also include additional questions to assess how HIVRR+MF participants have utilized the vocational training and financial literacy training that they have received.

To complement self-reports and utilize an objective assessment measure of risk behavior, we use biological assays to confirm HIV and STI status. We selected five STIs: syphilis, gonorrhea, chlamydia, trichomoniasis and mycoplasma as objective proxies of sexual risk behavior. The choice of these STIs was based on their high prevalence and incidence in this population and the availability of definitive treatment with a single-dose medication (which obviates STI medication treatment adherence and compliance issues). We also selected HCV to index drug-related risk behaviors. A clinical coordinator conducts biological testing in each project field office location. A complete description of test systems used is provided in Table 2.

**Table 2:** Biotesting systems used

Infection	Testing specimen	Test system used	Sensitivity (95% CI^a^)	Specificity (95% CI^a^)
HIV	Finger stick (whole blood sample)	Alere Determine™ HIV-1/2 Ag/Ab Combo rapid test system [47]	99.9% (99.4–100.0%)	99.8% (99.5–99.9%)
Hepatitis C (HCV)	Dried blood spot	Murex anti-HCV (version 4.0) [48]	100% (94.79-100%)	99.88% (99.77–99.94%)
Syphilis	Dried blood spot	Murex ICE Syphilis [49]	100% (99.1–100%)	100% (99.2–100%)
Gonorrhea	Vaginal specimen	AmpliSense *N. gonorrhea*/*C. trahomatis*/*M. genitalium*/*T. vaginalis*-MULTIPRIME-FRT PCR [50]	N/A^b^	N/A^b^
Trichomoniasis	Vaginal specimen	AmpliSense *N. gonorrhea*/*C. trahomatis*/*M*. *genitalium*/*T. vaginalis*-MULTIPRIME-FRT PCR [50]	N/A^b^	N/A^b^
Chlamydia	Vaginal specimen	AmpliSense *N. gonorrhea*/*C. trahomatis*/*M. genitalium*/*T. vaginalis*-MULTIPRIME-FRT PCR [50]	100% (73.3–100%)	100% (98.1–- 100%)
*Mycoplasma genitalium*	Vaginal specimen	AmpliSense *N. gonorrhea*/*C. trahomatis*/*M. genitalium*/*T. vaginalis*-MULTIPRIME-FRT PCR [50]	76.5% (50.1–93.0%)	100% (98.1–100%)

^a^CI confidence interval

^b^N/A estimates are not available for vaginal specimen samples

Participants who test positive for any STI are provided with referrals and treatment. The clinical coordinator works with individual participants to ensure that they complete their course of treatment prior to randomization and session 1, ensuring that any STI acquired post randomization is an incidence STI, and, as soon as possible at follow-up, biotesting assessments. STIs acquired and treated between the baseline and month-9 assessment or between the month-9 and month-15 assessment at an external clinic are only included as an endpoint if it is self-reported by participants on their month-9 and month-15 assessments. The clinical coordinator refers participants who screen positive for syphilis to the local Skin and Venereal Disease Dispensary to receive additional confirmatory testing for syphilis, consultations with a physician, and treatment for active forms of syphilis. Treatment for HCV is not widely available in Kazakhstan, and clinical coordinators refer participants who test positive for HCV to specialized clinics for additional examination, monitoring, and treatment. Treatment for HIV is also limited in Kazakhstan. If participants receive a positive rapid test for HIV, the clinical coordinator refers them to the local AIDS center for confirmatory testing and treatment. HIV-positive participants sign an additional waiver allowing our study access to treatment records at the local HIV clinic. Clinical coordinators collect information on confirmatory testing (for newly diagnosed cases of HIV), viral-load test results, CD4 test results, and antiretroviral (ARV) medication history at every assessment period for each participant.

Participants receive the equivalent of US$10 for the baseline assessment, US$9 for the month-6 follow-up assessment, US$11 for the month-9 follow-up assessment, and US$16 for the month-15 follow-up assessment. The total possible reimbursements for assessments is US$46.

#### Qualitative assessment

To help us open the “black box” of HIVRR+MF delivery, we conduct semi-structured, in-depth interviews at three points in time during study implementation: at 1 month, 3 months and 15 months post randomization and session 1 (corresponding to the end of the financial literacy sessions, vocational trainings sessions for HIVRR+MF participants, and to the month-15 follow-up assessment for all participants). We randomly select one participant from each cohort (approximately 50 women) to complete interviews. Questions focus on participants’ perception of the intervention, their experience with both the HIV-risk reduction and microfinance components and their reaction to the various role-plays, activities and goal-setting assignments, as well as the reactions or interactions with family, friends, colleagues related to their participation, factors promoting the use of the session information and skills and barriers impeding participation. Importantly, questions also focus on perceptions of how the intervention influenced participant safety, including any policy involvement, stigma or discrimination, drug use before and during the intervention, and savings and the potential access to matched savings, and alternative income sources. All interviews are audio-taped, transcribed and translated to English.

Cost-effectiveness is measured through a series of staff questionnaires, asking project staff about the time they spend on various intervention activities, along with budgets and financial records.

### Data analysis

In assessing primary outcomes (STI, HIV, and HCV incidence, unprotected sexual acts and unsafe injection acts), our over-arching modeling approach estimates intervention differences (HIVRR alone versus HIVRR+MF) longitudinally using a generalized estimating equations (GEE) approach, a flexible extension of conventional regression models, to account for repeated measures and clustering imposed by randomizing individuals in groups of six to eight women per cohort. STIs will be summarized as both a count (number of positive test results for any of the five STIs of interest (syphilis, gonorrhea, chlamydia, trichomoniasis, mycoplasma)) over the entire follow-up period, as well as a binary indicator of a positive test for any STI of interest at each follow-up time. The primary analysis will be a logistic GEE (e.g., presence/absence of an incident STI post randomization), accounting for clustering imposed by the study design in having women receiving their randomized intervention in cohorts of six to eight women each. Additionally, secondary analyses will consider intervention effects on HIV and HCV incidence as operationalized by binary indicators of testing positive for either, respectively, over the entire follow-up. We will employ logistic (binary outcomes) and Poisson (count outcomes) GEE models to estimate intervention effects as appropriate. We will implement an intent-to-treat approach for all analyses. Model estimates will be presented as estimates of incidence risk ratios or odds ratios, as appropriate, and corresponding 95% confidence intervals (CIs) and *p* values will be calculated for inference. Additionally, a logistic GEE modeling approach will be implemented for dichotomous outcomes (e.g., GEE with specified logit link) when evaluating the following outcomes: (1) self-reported unprotected vaginal and anal sexual acts with both regular and paying partners and (2) self-reported unsafe injection acts. Again, we will be interested in estimate rate ratios or odds ratios comparing HIVRR+MF to HIVRR alone. A Poisson GEE modeling strategy (e.g., GEE with specified log link) will be implemented to evaluate remaining secondary outcomes (1) self-reported number of unprotected vaginal and anal sexual acts with both regular and paying partners and (2) self-reported number of unsafe injection acts. To evaluate the secondary aims of the study related to potential moderating effects, we will expand these models to include “moderator X intervention” interaction terms along with the corresponding main effects and will assess examine the significance of each moderator on intervention effects through these interaction terms.

To examine whether key, theory-driven variables mediate the intervention’s effects on the primary outcomes, again we expand the logistic GEE modeling framework from the primary aim to examine (1) whether the HIVRR+MF improves the primary outcome compared to HIVRR alone; (2) whether the intervention improves each mediator; and (3) whether improvements in the mediator over time are associated with improvements in the primary outcome over time.

#### Power and sample size

Power and sample size considerations for this proposal prioritized sufficiently powering the study to detect intervention effects on incidence for the following biological outcomes: STIs (syphilis, gonorrhea, trichomoniasis, chlamydia, and mycoplasma), HIV and hepatitis C virus (HCV). Using baseline prevalence estimates from our prior work in similar populations (e.g., 10%, 28%, and 75%, for STIs, HIV, and HCV, respectively) [29], we conservatively focused on the least prevalent of the three biological outcomes of interest, STIs. We noted that if we were sufficiently powered to estimate intervention efficacy for the least prevalent outcomes, we would also be sufficiently powered to detect intervention effects for the others. Using these prevalence estimates, we determined a range of plausible incidences based on our experience in similar settings. We first calculated crude (unadjusted for clustering by cohort) using a conventional approach to detect differences in a binary outcome (e.g., incidence) in two groups assuming an *α*-level of 0.05 for a two-sided hypothesis test of an overall intervention effect. Crude sample size estimates were then inflated by the “variance inflation factor” as described in Donner and Klar [30], to account for the likely correlation of outcomes induced by the study’s cluster randomized design. Finally, we adjusted 20% attrition over the 15-month follow-up. We considered scenarios of modest (intraclass clustering coefficient (ICC) equal to 0.005) and moderate (ICC = 0.01) clustering effects. In summary, we determined that randomizing 350 women in equal proportions to each intervention (175 to HIVRR and HIVRR+MF) would ensure that 80% power to detect true relative risks of 0.25 and ICC = 0.01. This attrition and clustering adjusted sample size corresponds to detecting STI incidence relative risks (RR) equal to 0.25 comparing women randomized to HIVRR+MF to HIVRR alone, where the assumed STI incidence over the study follow-up period is 0.043 and 0.144, respectively. In these calculations, the assumed incidence in HIVRR alone of 0.144 represents a decrease of approximately 40% from the estimated baseline prevalence of 28% from our prior work in this population [29]. Because the other biological outcomes of interest are more prevalent, we expect to have greater than 80% power to detect similar intervention effects.

#### Qualitative analyses

Data from the in-depth interviews will be transcribed and translated prior to content analysis. Content analyses will identify emergent themes related to participants’ experiences with the intervention components that each received, how they utilized the skills learned in the intervention, and the impact that participation in the intervention had on their lives. Data will be analyzed using NVivo software, aided by group discussions to reach consensus on themes identified.

#### Cost-effectiveness analyses

Cost-effectiveness of the intervention will be analyzed based on STI infections and number of HIV and HCV cases averted over the 12-month period. We will compare cost-effectiveness of HIVRR+MF to HIVRR, using the cost of staff time, supplies, and overhead for the HIVRR and the MF interventions. We will calculate the incremental cost-effectiveness ratio between treatment *i* (HIVRR + MF) and *j* (HIVRR) in terms of outcome *k* by comparing extra cost with extra effectiveness in paired combinations: *ICERijk* = (*Ci* – *Cj*)/(*Eik* – *Ejk*) where *Ci* and *Cj* are the costs and *Eik* and *Ejk* are the effectiveness measures for treatments *i* and *j* measured along output *k*. This approach safeguards validity against any biased measurements of cost or effectiveness that are common to both *i* and *j* because they cancel out. Policymakers can easily and directly compare *ICERijk* to their existing costs and cost-effectiveness metrics to assess the added benefit of the microfinance intervention. We will also report total cost-effectiveness ratios for each intervention: *CERik* = *Ci/Eik*. To construct an effectiveness outcome of HIV, STIs, and HCV transmissions averted, we will use standard modeling procedures reported in HIV cost-effectiveness literature; i.e., a Bernoulli Process Model, the difference in risk-behavior frequencies times HIV transmission probabilities per act [31, 32]. One-way sensitivity analyses will use a range (e.g., 95% CIs) of HIV and HCV rates in the target population that may be compared to results to assess the robustness of estimates. Policymakers may compare the HIV, STIs, and HCV cost-effectiveness ratio to ratios for existing HIV interventions [31, 32] to ascertain the potential value of the microfinance intervention.

### Quality assurance and fidelity

Standardized protocols and trainings are used to assure consistency and fidelity to the intervention and assessments across multiple study sites. Research assistants, clinical coordinators, and facilitators receive standard training in their areas of responsibility, including procedural manuals and checklists. The use of CASI for baseline and follow-up behavioral assessments ensures that each participant is receiving the same version of the questionnaire, and that there is no response bias. Each site receives daily supervision from a site coordinator, who is responsible for reviewing study records and reporting any irregularities or protocol violations to the study team. Data management staff on the study team are responsible for coordinating data collection across multiple databases. Weekly Skype meetings with study investigators are used to address any issues or challenges.

A Data Safety and Monitoring Board (DSMB), consisting of Kazakhstan-based researchers, has been identified and meets on an annual basis to review all ongoing study data and human subjects issues.

## Discussion

This paper describes the protocol for implementing a combination HIV-risk reduction and savings-led microfinance intervention, conducted among high-risk women in Kazakhstan. This clinical trial has a number of strengths, including its understudied and underserved target population of women who both use drugs and engage in sex trading for drugs and money, its selection of a combination HIVRR and a savings-led microfinance intervention as a structural intervention for these women, and the implementation of this intervention through a rigorous study design.

As we have described above, the specific target population of our intervention has been largely absent in past HIV-risk-reduction interventions. While combination HIVRR and microfinance interventions have been shown to reduce HIV risk and empower women to protect themselves, these studies rarely focus on women who use or inject drugs. Both our intervention and our assessments are tailored to the needs and realities of women who share the dual risks of sex work and drug use. HIVRR intervention sessions carefully incorporated core components of the intersection of drug use and sexual risk, and risk-reduction strategies with paying partners. We also structured the behavioral questionnaires to address constructs that have never been scientifically evaluated among this high-risk group in Kazakhstan; e.g., experience of violence by FSW [2].

The intervention presented in this study is one of the few which has combined HIVRR with savings-led microfinance, and the only one to do so among this target population. This trial, therefore, meets the call for more structural interventions for HIVRR which are designed for women who use drugs [22] and who sell sex [33]. The HIVRR intervention sessions not only address women’s behavioral risks for HIV, but also the complex risk environment faced by our participants, through overdose response training, service referrals and safety planning. The microfinance component of the program further builds on these risk environment elements by addressing women’s economic empowerment. The selection of a savings-led microfinance was based on the limitations that microcredit and microloan programs pose [34]. Our savings-led approach enables participants to accumulate assets faster and pay for life-cycle events without accumulating debt and an overreliance on microloans.

Finally, our study addresses many of the scientific limitations of previous studies.

Ours is the first RCT conducted with FSW in Kazakhstan, and one of the few conducted in Central Asia. Our use of a longitudinal study design, assessing outcomes over a 12-month post-intervention period, allows for us to examine the long-lasting effects of our intervention and assess the sustainability of its outcomes. Our mixed-methods approach, which includes biological assessments, behavioral questionnaires, in-depth interviews, and cost data, allows us to assess the impact of our intervention on multiple levels. The qualitative component of our study uses in-depth interviews to probe deeper into the barriers to savings and vocational entry experienced by FSW, as well as providing information on the feasibility and acceptability of the intervention.

Academic literature on microfinance for HIV-risk reduction has called for a more rigorous testing of interventions, examination of the causal pathways between microfinance and reduced risk-behavior outcomes, and the use of biomarkers [12, 14]. Our assessments provide much-needed data on the experiences, risks and needs of this population in Kazakhstan, the variables that moderate and mediate HIV-risk outcomes, and how these differ between treatment and control conditions. We are the first study, to our knowledge, to use biological testing to supplement self-reported HIV and STI data. Our stringent quality control measures and the use of a computerized data collection tool ensure standardization and consistency among participants.

### Challenges and limitations

Our study design uses several tools for reducing biases. Cluster randomization methods may offset the effects of selection bias, and analyses will compare baseline characteristics of both treatment and control arms. Our assessment measures include sensitive information on highly stigmatized behaviors, including sexual behavior history and drug use. We recognize the risk of reporting bias in these assessments, and have taken measures to minimize the risk of this, including establishing trust through the informed consent process and the use of CASI, which allows participants privacy and anonymity as they complete the survey measures. Finally, given the long time frame of our study and the staggered start dates between the Temirtau and Almaty sites, we suspect that external confounding factors may influence participant enrollment and intervention outcomes. For example, we have already observed a rapid contraction of Kazakhstan’s economy and a currency devaluation in the summer of 2015, which may impact participants’ savings behavior.

We expect several challenges in recruitment and retention of participants. FSW who use drugs are highly marginalized, and often hesitant to seek healthcare and other services for fear of stigma or legal repercussions [5]. Our decision to recruit at field locations as well as through a peer-referral system was intended to extend the reach of our study to as many FSW as possible. However, there is some evidence that shows that network-based recruitment and venue-based recruitment of FSW yields populations with different sociodemographic characteristics and even different STI prevalence [35]. We aim to resolve this issue through random assignment to study condition, and to conduct analyses that consider recruitment source, and any other differences potentially identified between study conditions, as a moderating variable.

We have instituted a number of strategies to address potential loss to follow-up of participants. Past studies have found that up to 80% of FSW move between cities [2], and our selection of Almaty as a study site, a border town with high rates of migration and mobility, suggests that attendance at follow-up recruitment may be low. We have tried to preemptively address this problem through: a month-long follow-up window, which allows for maximum flexibility, the collection of exhaustive contact information for each participant; the use of retention visits for control cohorts to maintain contact during their longer post-intervention period prior to the first follow-up; and through the creation of a supportive and pleasant office and intervention group atmosphere, which, we hope, will encourage women to return.

### Implication of this study in HIV prevention

Our study examines the benefits of investment in income-generating interventions as an HIV prevention strategy among high risk women. If successful, it may remove some of the hesitations and stigma that prevent investment in economic opportunities for women who use drugs or who sell sex. We hope that this intervention model will demonstrate that HIV prevention for women who use drugs in Central Asia and other countries worldwide can benefit from a combination of HIV-risk reduction and economic empowerment. We expect to see it implemented at the local and national level in Kazakhstan, as well as in other counties in Central Asia and globally in countries where drug use among women is prevalent.

### Trial status

Recruitment for this study began in May 2015, and the last cohort of participants was enrolled in July 2017. Follow-up assessments will continue through October 2018.

## Additional file

Additional file 1: SPIRIT 2013 Checklist: Recommended items to address in a clinical trial protocol and related documents. (DOC 123 kb)

## Abbreviations

CASI: Computer-assisted self-interview
c-RCT: Cluster randomized controlled trial
FLT: Financial Literacy Training
FSW: Female sex worker
GBV: Gender-based violence
HCV: Hepatitis C virus
HIVRR: HIV-risk reduction
IPV: Intimate-partner violence
MF: Microfinance
NGO: Non-governmental organization
RCT: Randomized controlled trial
